# The prognostic value of circulating cell-free DNA in breast cancer: A meta-analysis

**DOI:** 10.1097/MD.0000000000010197

**Published:** 2018-03-30

**Authors:** Guoqiang Tan, Chang Chu, Xiujuan Gui, Jinyuan Li, Qiufang Chen

**Affiliations:** a Department of Central Laboratory; b Department of Nephrology, The First Affiliated Hospital of Jinan University, Guangzhou, Guangdong Province, People's Republic of China.

**Keywords:** breast cancer, circulating cell-free DNA, meta-analysis, survival outcome

## Abstract

**Background:**

Circulating cell-free DNA (cfDNA) isolated from plasma or serum by noninvasive procedures can serve as a “liquid biopsy” and has potential as a biomarker for the tumor burden and survival prediction of breast cancer (BC). However, its prognostic value in patients with BC is currently under debate. The aim of this meta-analysis was to investigate the relationship between cfDNA and survival outcome.

**Methods:**

We systematically searched *PubMed*, *Embase*, and *Science Citation Index* electronic databases for studies about the prognostic utility of cfDNA in patients with BC. The clinical characteristics, relapse/disease-free survival (RFS/DFS), and overall survival (OS) data were extracted from the eligible studies. The hazard ratios (HR) and 95% confidence intervals (CI) were calculated and pooled with a fixed-effects model using the Stata12.0 software. Subgroup and sensitivity analyses were also performed.

**Results:**

This meta-analysis included a total of 10 eligible studies and 1127 patients with BC. The pooled HR with 95% CI showed strong associations between cfDNA and OS (HR = 2.41, 95% CI, 1.83–3.16) along with DFS/RFS (HR = 2.73, 95% CI, 2.04–3.67) in patients with BC. Although publication bias was found in the studies regarding RFS/DFS, further trim and fill analysis revealed that the adjusted HR would be 2.53 (95% CI, 1.83–3.51), which is close to the original HR. Subgroup analyses confirmed the role of cfDNA as a strong prognostic marker in patients with BC, regardless of cfDNA analysis, sampling time, sample source, detection method, tumor stage, sample size, or area.

**Conclusions:**

Our meta-analysis indicates that cfDNA is a strong predictive and prognostic marker in patients with BC.

## 1 Introduction

Breast cancer (BC) is the most frequently diagnosed malignancy and the leading cause of cancer death among women worldwide.^[1]^ Although early detection and comprehensive treatment have been implemented, the 5-year survival rate among patients with BC who develop metastatic disease is only 24%.^[2]^ To improve the clinical outcome of patients, there is an urgent need for the development of early biomarkers that could provide prognostic information regarding treatment response and disease progression.

At present, evaluation of the metabolic tumor burden in BC is dependent on serum markers, including Cancer Antigen 15-3 (CA15-3) and Carcinoembryonic Antigen (CEA). The major issues with using these markers, however, are a *lack of sensitivity and specificity*, especially in early or localized disease.^[3,4]^ Therefore, in recent years, much effort has been devoted to the detection and characterization of circulating cell-free DNA (cfDNA) in BC. cfDNA is nucleic acid fragments that circulate in the plasma, serum, and other bodily fluids outside of cells. The first report was by Mandel and Metais in 1948,^[5]^ but the topic attracted little attention in the scientific community. It was not until 1977 that cfDNA was recognized as a useful tool for monitoring the effects of therapy in cancer patients.^[6]^ In healthy individuals, cfDNA is derived from apoptotic cells and is truncated, measuring 185 to 200 base pairs (bp) in length, whereas in cancer patients it results from apoptotic and necrotic cells.^[7,8]^ Therefore, elevated levels of long DNA fragments may be a good marker for the detection of malignant tumor DNA in blood.^[7,9]^ Recent evidence showed that DNA integrity, the ratio of longer to shorter DNA fragments, was significantly higher in relapsed patients with BC than in patients who were free of disease.^[10]^ Furthermore, tumor-associated genetic alterations, such as single nucleotide variants (SNVs), copy number alterations (CNA), and structural variants (SVs), can be detected in cfDNA termed circulating tumor DNA (ctDNA).^[11]^ Several studies on BC have investigated the prognostic value of cfDNA for detecting PIK3CA/p53 mutation and DNA methylation.^[12–15]^ The results suggested that the genotype of cfDNA could be a promising tumor biomarker for BC. The variability in levels of cfDNA cannot be definitively determined, but is closely related to the tumor burden and response to therapy.^[14–18]^

Although current research progress involving cfDNA is encouraging, the prognostic relevance of cfDNA in BC remains controversial. Two meta-analyses addressing the controversies regarding the prognostic value of cfDNA in nonsmall cell lung cancer and colorectal cancer have recently been published.^[19,20]^ However, no meta-analyses have yet reported the predictive and prognostic value of cfDNA in patients with BC. Therefore, a meta-analysis designed to address this topic was conducted.

## 2 Methods

### 2.1 Search strategy

We searched the studies in 3 electronic databases: *PubMed*, *Embase*, and the *Science Citation Index* up to January 2017 without applying start a date limit. The terms BC, mammary cancer, cell-free DNA, plasma DNA, serum DNA, and prognosis were searched as MeSH and free words at the same time. First, unrelated studies were excluded by reading the titles, author details, and abstracts. Duplicate or non-English articles, review articles, letters, and other ineligibility types of articles were also excluded. Finally, the full texts of each remaining potential article were reviewed to examine whether articles met the eligibility criteria.

### 2.2 Criteria for inclusion and exclusion

The inclusion criteria for eligible studies were as follows: patients were diagnosed with BC; cfDNA was isolated from plasma or serum in the peripheral blood rather than tumor tissue; the hazard ratio (HR) and corresponding 95% confidence interval (CI) for survival outcome were directly provided or could be statistically estimated, when several studies reported on the same group of patients, the latest study was included; and the study was designed as a cohort study.

The exclusion criteria were as follows: lack of sufficient survival data; inability to obtain the full text; non-English articles; reviews, letters, case reports, conference abstracts, and duplicate articles; and small-sample studies with fewer than 30 patients.

### 2.3 Quality assessment of studies

The quality of the eligible studies in this present meta-analysis was assessed independently by 2 reviewers according to the Newcastle–Ottawa Scale (NOS). The NOS includes 3 broad aspects, allowing the evaluation of the studies by patient selection, study comparability, and outcome of interest. These aspects yield maximum scores of 4, 2, and 3, respectively, for a total score of up to 9. Studies with scores not <5 are considered high quality; studies with lower scores were removed from this meta-analysis because of low quality. Disagreements on the quality assessment of studies were solved through discussion.

### 2.4 Ethical statement

All analyses were from previous published studies, and not involved human being and experimental subjects. Therefore, no ethical approval was required.

### 2.5 Data extraction and statistical analysis

Two reviewers independently extracted the following data from each eligible study: surname of the first author, publication year, country, clinical stage, patient number, sampling time, sample source, methods of cfDNA detection, and follow-up time. We also extracted survival data including relapse/disease-free survival (RFS/DFS), overall survival (OS), and the HR and its 95% CI.

The prognostic value of cfDNA in this meta-analysis was performed using the pooled HR and its 95% CI. For each study, the HR and its 95% CI were either directly reported or estimated using the method suggested by Tierney et al.^[21]^ Heterogeneity between studies was evaluated using the Cochran *Q* test and the *I*^2^ test. Fixed-effect models were adopted only for a *P* > .1 or *I*^2^ < 50%. Otherwise, random-effect models were applied to calculate the pooled HR. To evaluate publication bias, a funnel plot, Egger test, and Begg test were used. If publication bias was observed in this meta-analysis, trim and fill analysis was conducted to evaluate the number of missing studies and recalculate the pooled risk estimate with the addition of those missing studies.^[22]^ Further subgroup analyses are necessary because of the potential factors that might influence the prognostic value of cfDNA in BC. All statistical analyses were conducted using the Stata version 12.0 software.

## 3 Results

### 3.1 Search results

**Figure 1:**
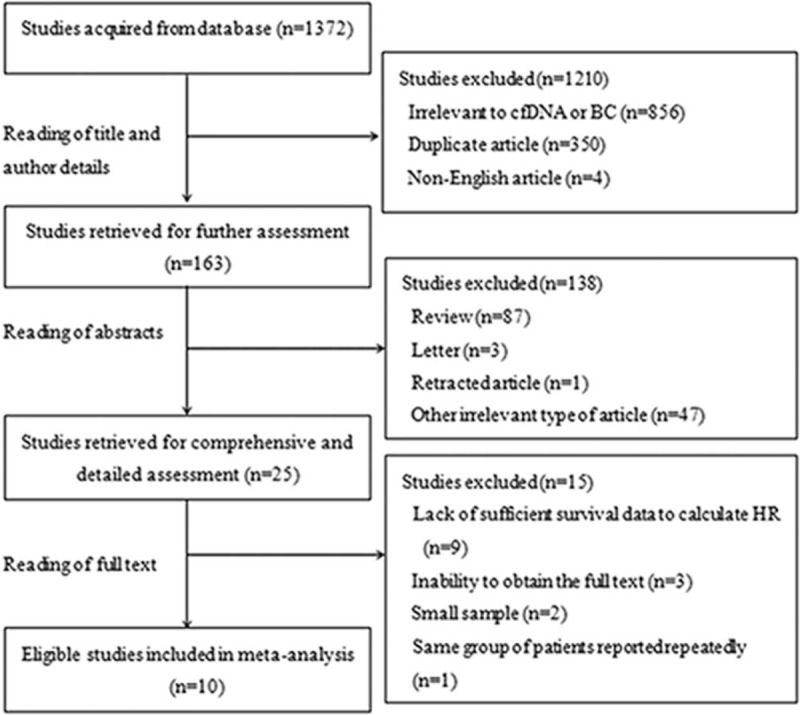
Flow chart of studies selection.

Based on the search strategy, a total of 1372 relevant studies were acquired from *PubMed*, *Embase*, and the *Science Citation Index* database. However, only 25 studies were further reviewed after reading the titles, author details, and abstracts. According to the criteria for inclusion and exclusion, 15 ineligible studies were excluded because they used small sample, the full text could not be obtained, sufficient survival data to calculate the HR were not available, or the same group of patients was repeatedly reported in a later study. Eventually, 10 eligible studies were included in this meta-analysis (Fig. 1)

### 3.2 Study characteristics

The 10 studies were published between 2001 and 2017, and a total of 1127 patients with BC from Asia (n = 6) and Europe (n = 4) were enrolled in the meta-analysis. Early-stage (I–III, n = 840) and metastatic (IV, n = 183) BC patients were enrolled in 6 and 2 studies, respectively. Another 2 studies had collected patients in stages I–IV (n = 104). Five studies assessed the prognostic value of cfDNA concentration in BC. The prognostic values of ctDNA and DNA methylation were each reported in 2 studies. In addition, TP53 and PIK3CA mutations in cfDNA were each analyzed in 1 study. The 1 remaining study assessed the prognostic value of cfDNA integrity in BC. All studies were considered high quality with NOS scores of 5 points or more (Table 1). The main characteristics of the eligible studies are shown in Table 2.

**Table 1:**
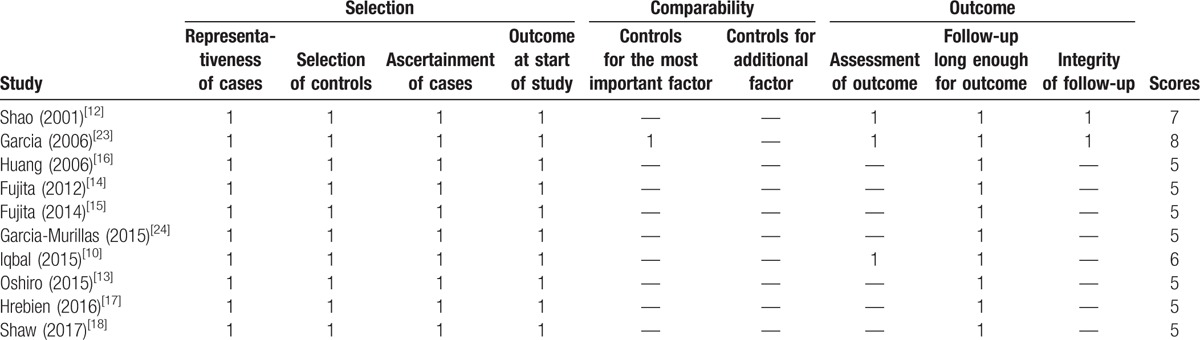
Newcastle–Ottawa Scale (NOS) for quality assessment in meta-analysis.

**Table 2:**
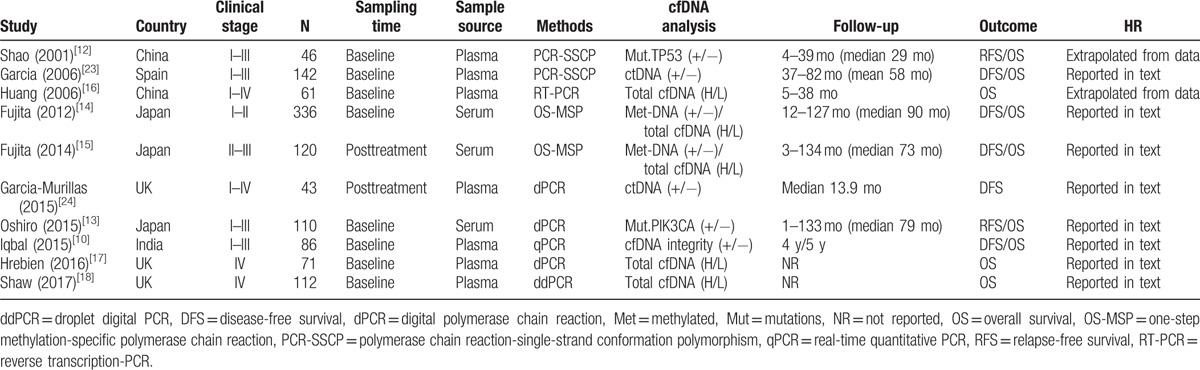
The main characteristics of eligible studies in the meta-analysis.

### 3.3 Meta-analysis of cfDNA to evaluate its prognostic value

**Figure 2:**
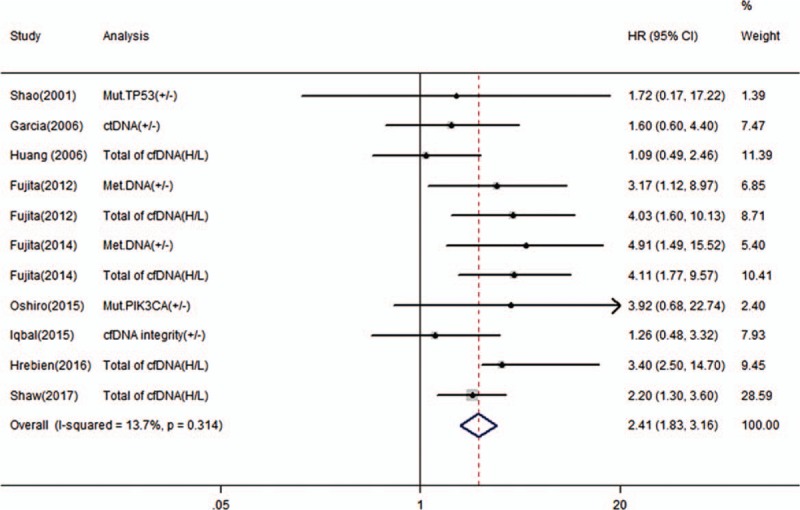
Forest plot of hazard ratios for correlations between cfDNA analysis and OS.

**Figure 3:**
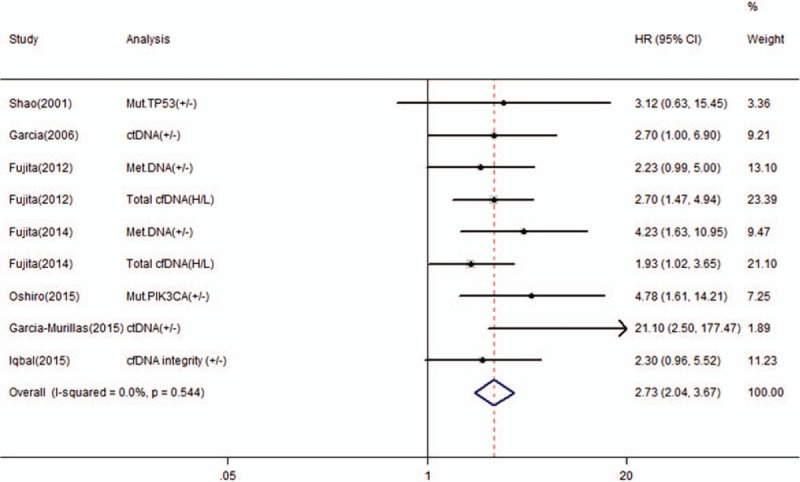
Forest plot of hazard ratios for correlations between cfDNA analysis and RFS/DFS.

Nine studies reported the correlation between cfDNA and OS in BC patients and 7 studies reported the correlation between cfDNA and RFS/DFS. We calculated pooled HR using a fixed-effects model because no heterogeneity was observed in this meta-analysis (*I*^2^ = 13.7%, *P* *=* .314 for OS; *I*^2^ = 0.0%, *P* *=* .544 for RFS/DFS). Overall, the pooled results indicated that the detection of cfDNA had significant value in predicting OS (HR 2.41, 95% CI, 1.83–3.16, *P* = .000) and RFS/DFS (HR 2.73, 95% CI, 2.04–3.67, *P* = .000) in BC patients (Figs. 2 and 3).

### 3.4 Publication bias

**Figure 4:**
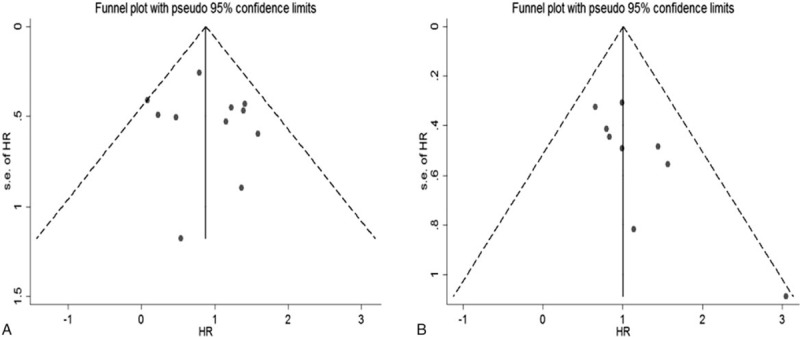
Funnel plots of publication bias for OS (A) and RFS/DFS (B) in present meta-analysis.

**Figure 5:**
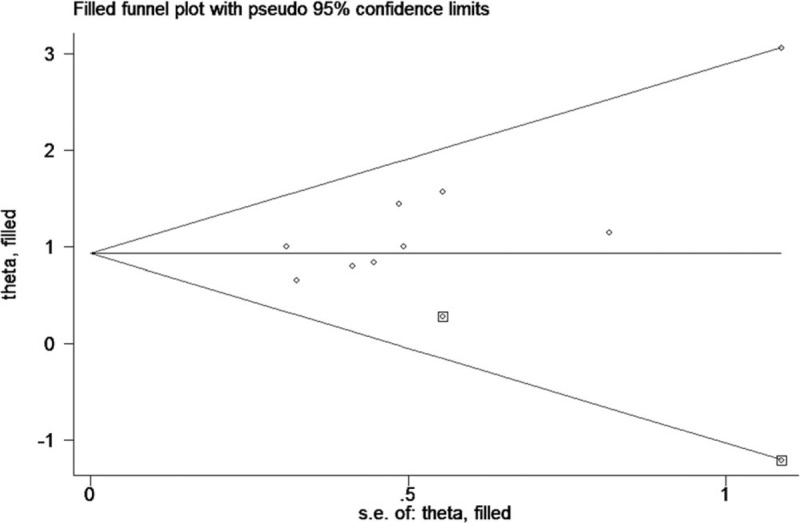
Funnel plots of trim and fill analysis for RFS/DFS in present meta-analysis.

The funnel plot of publication bias was symmetric for OS (Fig. 4B). Furthermore, no significant publication bias for OS was revealed by Egger test (*P* = .597) or Begg test (*P* = .755) in the this study. However, publication bias was found for RFS/DFS according to the asymmetric funnel plots, Egger test (*P* = .019) and Begg test (*P* = .016). Based on the funnel plots of trim and fill analysis for RFS/DFS, there are 2 missing studies were imputed in the contour-enhanced funnel plots (Fig. 5). The analysis indicated that the imputed HR was 2.53 (95% CI, 1.83–3.51), which was consistent with our original conclusion.

### 3.5 Subgroup analyses

Although no heterogeneity was observed in this meta-analysis for OS and RFS/DFS, recent studies suggest that marker type, marker origin, tumor stage, method, and sample size would have considerable impact on the relationship between the presence of cfDNA and the outcomes of cancer patients.^[20]^ Therefore, subgroup analysis was implemented based on the above factors. As shown in Table 3, the subgroup analyses indicated that cfDNA analysis might contribute to determining the clinical heterogeneity for OS. Meanwhile, stratification by sampling time and area might contribute to the clinical heterogeneity for RFS/DFS.

**Table 3:**
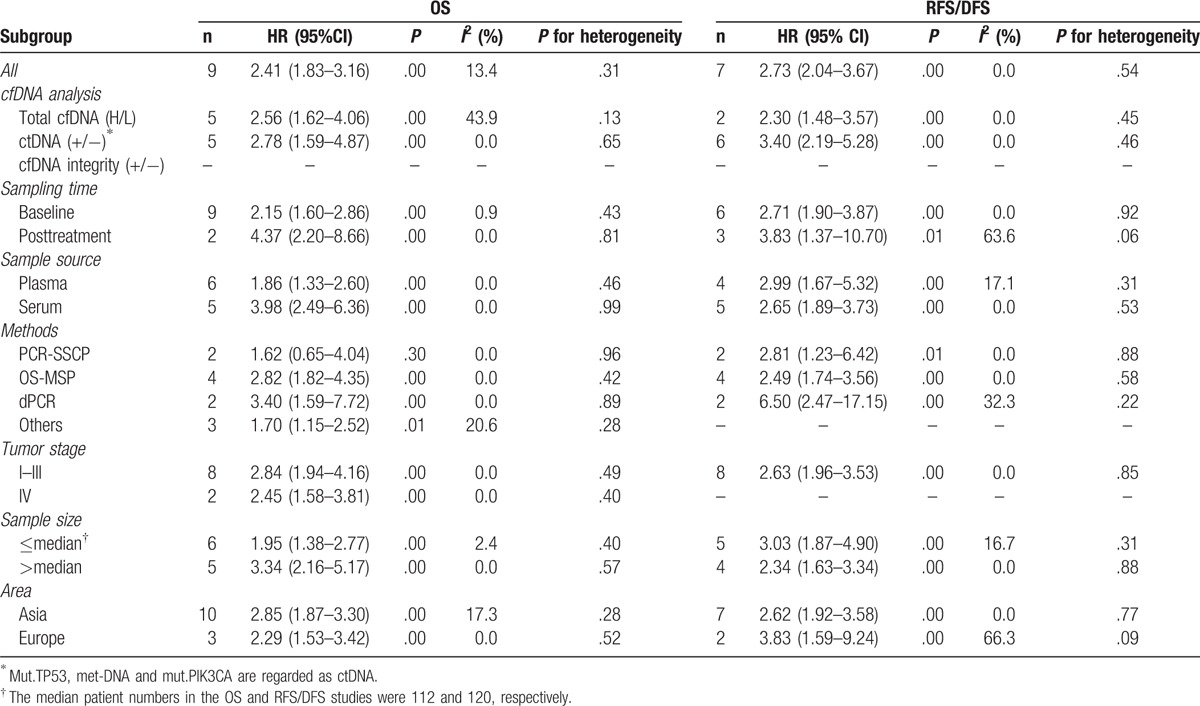
Results of subgroup analyses on OS and RFS/DFS studies.

For OS, the pooled HR of the ctDNA (+/−) subgroup was 2.78 (95% CI, 1.59–4.87, *P* = .00), which was higher than that of the total cfDNA with high or low levels (H/L). Mut.TP53, met-DNA, and mut.PIK3CA are regarded as ctDNA in our meta-analysis. Similarly, the pooled HR of the ctDNA (+/−) subgroup for RFS/DFS was 3.40 (95% CI, 2.19–5.28, *P* = .00), which was significantly higher than the value of 2.30 (95% CI, 1.48–3.57, *P* = .00) for the total cfDNA (H/L) subgroup, suggesting that ctDNA (+/−) might be more closely associated with poor survival in BC. Equally, when stratified by sampling time, the pooled HR of the posttreatment subgroup was significantly higher than that of the baseline subgroup for both OS and RFS/DFS. However, no similar behavior of the pooled HR was found in the subgroup analysis based on sample source. The pooled HR values of the dPCR subgroup were 3.50 (95% CI, 1.59–7.72, *P* = .00) and 6.50 (95% CI, 2.47–17.15, *P* = .00) for OS and RFS/DFS, respectively, which were significantly higher than the values for the other 3 subgroups. Finally, the results of subgroup analyses, categorized by tumor stage, sample size, and area, suggested that both the pooled HR and the corresponding 95% CI were >1.

### 3.6 Sensitivity analysis

**Figure 6:**
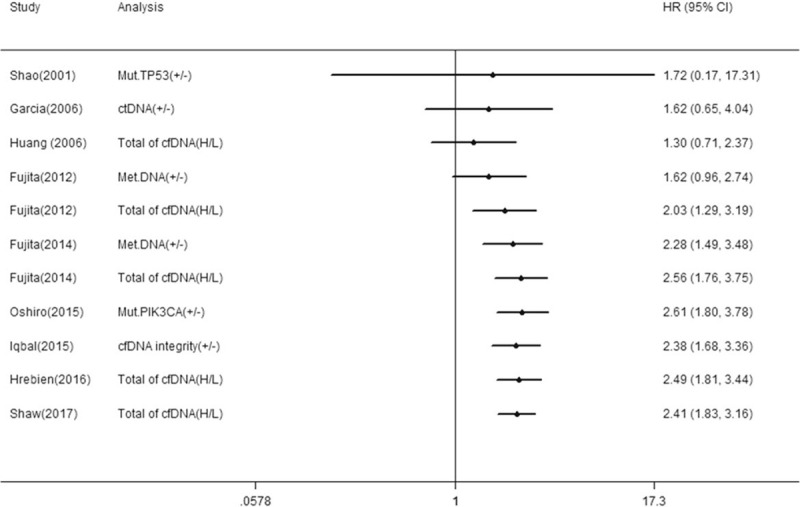
Cumulative meta-analyses of OS by publication year.

**Figure 7:**
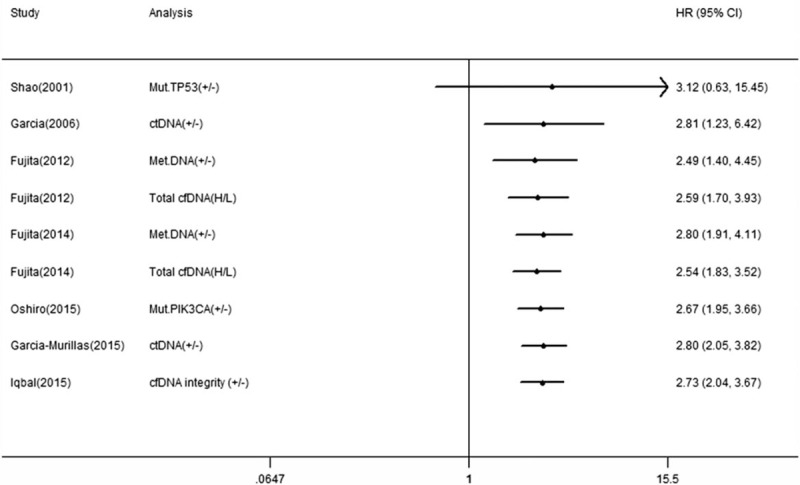
Cumulative meta-analyses of RFS/DFS by publication year.

To assess whether the results were reliable, it was necessary to perform sensitivity analysis. First, each individual study was randomly removed, and the pooled HR was recomputed. The pooled HR of these sensitivity analyses varied from 2.26 (95% CI, 1.70–3.02) to 2.66 (95% CI, 2.00–3.56) for OS, which showed no significant changes in overall effects for OS. Similarly, the pooled HR for RFS/DFS ranged from 2.61 (95% CI, 1.92–3.56) to 3.00 (95% CI, 2.16–4.18), which also showed no significant changes in overall effects for RFS/DFS. We further performed cumulative meta-analyses to determine the stability of cfDNA detection for survival in patients with BC (Figs. 6 and 7). With the inclusion of studies that published from 2001 to 2017, the pooled HR for OS ranged from 1.30 to 2.61. The pooled HR for RFS/DFS varied from 2.49 to 3.12 with the inclusion of studies that published from 2001 to 2015, indicating that the prognostic value of cfDNA detection for survival in patients with BC was stable.

## 4 Discussion

This meta-analysis demonstrated that the presence of cfDNA could be used to predict OS and RFS/DFS in patients with BC. The pooled HR values were 2.41 for OS and 2.73 for RFS/DFS in patients with BC. Subgroup analyses revealed that the pooled HR and its 95% CI in each subgroups were >1. The funnel plot, Begg test, and Egger test confirmed the absence of significant publication bias in this study for OS, but not for RFS/DFS. Based on trim and fill analysis, the adjusted HR of RFS/DFS was 2.53, which is close to the original HR. Furthermore, sensitivity analysis and cumulative meta-analyses demonstrated that the predictive value of cfDNA detection for the prognosis of BC patients was reliable.

Over recent decades, growing evidence has supported the potential role of circulating tumor cells (CTCs) and cfDNA as “liquid biopsies” to detect tumor progression and therapeutic response in real time.^[25]^ Several meta-analyses have previously reported the detection of CTCs as a stable prognosticator in patients with BC.^[26–28]^ Even the eighth edition of the AJCC TNM staging system for BC affirmed that if there is no clinical or imaging evidence of distant disease, but there is molecular or microscopic evidence of CTCs, then the cancer is classified as cM0 (i+).^[29]^ However, to the best of our knowledge, this is the first comprehensive meta-analysis to confirm the prognostic role of cfDNA in patients with BC, including qualitative and quantitative analysis of cfDNA. A high level of total cfDNA and the presence of ctDNA in the peripheral blood were significantly associated with poor prognosis in BC patients. Compared with a high level of total cfDNA, the detection of ctDNA may more effectively predict survival outcome, as the pooled HR of the ctDNA (+/−) group was higher than that of the group with a high level of total cfDNA. This result was similar for OS and DFS/RFS. Although the impact of ctDNA is an overall one and impact of single mutation might have less power. Similarly, we observed that all detection methods were effective for the detection of cfDNA, whereas the pooled HR seems to be more prominent in the dPCR subgroups than in the other 3 subgroups. The reason might be that the dPCR method was more sensitive and specific than the other methods. Considering that sampling time might be a source of clinical heterogeneity and affect the pooled HR, we compared the baseline and posttreatment values. The comparison results demonstrated that the pooled post-treatment HR values were significantly higher than those of the baseline subgroup for both OS and DFS/RFS, suggesting that the prognostic significance of cfDNA for BC patients when detected post-treatment was stable and reliable. In this meta-analysis, the cfDNA was extracted from the plasma or serum. Further subgroup analysis classified by sample source showed that cfDNA could be a predictive and prognostic marker in BC patients. The subgroup analysis classified by tumor stage showed that cfDNA was applicable to both early-stage and metastatic groups of BC patients. The pooled results are fairly stable and are not influenced by sample size and area. Finally, cfDNA analysis type, sampling time, method, and area might contribute to the substantial interstudy heterogeneity of the included studies.

This meta-analysis suggested a prognostic value of cfDNA in predicting the outcome of patients with BC, but several limitations should be considered. First, our meta-analysis is based on individual unadjusted HR values from studies whose results have been published, not from individual patient data. This data source may lead to lack some of accuracy and persuasiveness. Second, 2 studies do not report HR directly, and we had to calculate these values from the given data. Although we followed the procedure recommended by Tierney et al,^[21]^ the resulting HR and its 95% CI may be inaccurate. Third, only 1 study focused on cfDNA integrity. Thus, determining whether cfDNA integrity could serve as a powerful biomarker with prognostic value for BC patients will require more large and prospective studies. Moreover, the gray literature was not included in the meta-analysis. Generally, smaller samples were enrolled in gray literature, which showed an overall worse treatment effect than published trials,^[30]^ and our meta-analysis had a potential risk of overestimating the prognostic role of cfDNA in BC patients.

## 5 Conclusions

In summary, our meta-analysis indicates that cfDNA is a strong predictive and prognostic marker in BC. Both high levels of cfDNA and the presence of ctDNA were significantly associated with poor DFS/RFS and OS in patients with BC. Further large clinical trials are required to confirm our conclusion, which might help to define high-risk patients and guide personalized treatment in cancer patients.

## Author contributions

**Conceptualization:** G. Tan.

**Data curation:** G. Tan, X. Gui.

**Formal analysis:** G. Tan, X. Gui.

**Investigation:** G. Tan, C. Chu, X. Gui.

**Methodology:** G. Tan, J. Li.

**Project administration:** J. Li.

**Resources:** J. Li.

**Software:** G. Tan, Q. Chen.

**Supervision:** G. Tan, Q. Chen.

**Validation:** G. Tan, Q. Chen.

**Visualization:** G. Tan.

**Writing – original draft:** G. Tan, C. Chu.

**Writing – review & editing:** G. Tan, C. Chu.

## Acknowledgments

We thank Jinan University that provides a broad stage for us. Finally, Guoqiang Tan wants to thank, in particular, the patience, care, and encouragement from Chang Chu in the past 3 years.
